# Molecular Analysis of *TTF-1* and *TTF-2* Genes in Patients with Early Onset Papillary Thyroid Carcinoma

**DOI:** 10.1155/2012/359246

**Published:** 2012-03-11

**Authors:** Carla Espadinha, Ana Luísa Silva, Rafael Cabrera, Maria João Bugalho

**Affiliations:** ^1^Centro de Investigação de Patobiologia Molecular, Instituto Português de Oncologia de Lisboa Francisco Gentil E.P.E., 1099-023 Lisboa, Portugal; ^2^Serviço de Anatomia Patológica, Instituto Português de Oncologia de Lisboa Francisco Gentil E.P.E., 1099-023 Lisboa, Portugal; ^3^Serviço de Endocrinologia, Instituto Português de Oncologia de Lisboa Francisco Gentil E.P.E., 1099-023 Lisboa, Portugal; ^4^Clínica Universitária de Endocrinologia, Faculdade de Ciências Médicas, Universidade Nova de Lisboa, 1169-056 Lisboa, Portugal

## Abstract

Two common variants, close from *TTF-1* and *TTF-2*, were shown to predispose to thyroid cancer (TC) in European populations. We aimed to investigate whether *TTF-1* and *TTF-2* variants might contribute to TC early onset (EO). Tumor samples from eighteen patients with papillary TC (PTC), who underwent total thyroidectomy at an age of ≤21, were screened for *TTF-1* and *TTF-2* variants. No *TTF-1* variants were documented; two novel germinal *TTF-2* variants, c.200C>G (p.A67G) and c.510C>A (p.A170A), were identified in two patients. Two already described *TTF-2* variants were also documented; the allelic frequency among patients was not different from that observed among controls. Moreover, *RET/PTC* rearrangements and the *BRAF*V600E mutation were identified in 5/18 and 2/18 PTCs, respectively. Thyroglobulin (TG) and thyroid peroxidase (TPO) expression was found to be significantly decreased in tumors, and the lowest level of TPO expression occurred in a tumor harboring both the p.A67G*TTF-2* variant and a *RET/PTC3* rearrangement.

## 1. Introduction

Thyroid cancer is not common among children and adolescents. Between 1975 and 2000, thyroid cancer represented approximately 7.8% of all cancers diagnosed in the 15- to 19-year age group according to data in the Surveillance Epidemiology and End Results (SEERs) database [1]. It is very rare in children younger than 15 years of age with very few cases diagnosed before 10 years of age. Differentiated thyroid cancer (DTC), that is, papillary (PTC) or follicular thyroid cancer (FTC), accounts for the vast majority of cases and medullary thyroid cancer (MTC) accounts for the most of the rest. Poorly differentiated and anaplastic cancers are exceedingly rare.

Among environmental risk factors, a well-known risk factor for PTC is exposure to ionizing radiation and for FTC is deficiency in iodine intake. Common variants on 9q22.33 and 14q13.3 were shown to predispose to thyroid cancer in European populations [2]. The gene nearest to the 9q22.33 locus is *FOXE1* (*TTF-2*), and *NKX2-1* (*TTF-1*) is among the genes located at the 14q13.3 locus. Both genes are important in the biology of thyroid gland, and their expression is altered in thyroid tumors [3, 4].

Based on the hypothesis that young patients are carriers of genetic variants that influence their early onset of disease, we analyzed samples from patients who developed thyroid cancer early in life. Besides screening for the classical somatic changes, known to cause neoplastic transformation, we sought for *TTF-1* and *TTF-2* variants.

## 2. Materials and Methods

### 2.1. Patient Samples

Eighteen primary thyroid tumor samples from patients who underwent total thyroidectomy at age ≤ 21 years (mean age, 14,1 ± 4,4 yr; range, 5–21 yr), were analyzed. All were PTC. Exclusion criteria were prior exposition to radiation or family history of thyroid disease. Thirteen of these samples have already been included in a previous study [5]. In seven cases, it was possible to pair tumors with their normal adjacent tissue or contralateral lobe. Peripheral blood samples from patients and 32 healthy controls were also analyzed.

Tissue sample collection was carried out in accordance with protocols approved by the institutional review board, and informed consent was obtained for the study together with the consent for surgery.

### 2.2. DNA, RNA Extraction, and cDNA Synthesis

Total RNA was obtained from frozen tissues using TRizol reagent (Invitrogen, Paisley, UK), according to manufacturer's instructions, and 2 *μ*g were reverse transcribed using random primers and SuperScript II (Invitrogen). When applicable, genomic DNA was extracted from peripheral blood lymphocytes using Puragene DNA Purification System Blood Kit (Gentra, USA).

### 2.3. Screening for *BRAF* ^V600E^ Mutation and RET/PTC Rearrangements

Screening for the *BRAF* ^V600E^ mutation and *RET/PTC* rearrangements 1–3 was conducted as previously described [6].

### 2.4. TTF-1/NKX2-1 and TTF-2/FOXE1 Variants Screening

The mutational analysis of *TTF-1/NKX2-1* and *TTF-2/FOXE1* was performed in all tumor samples by RT-PCR followed by direct sequencing. Two transcript variants encoding different protein isoforms have been described for *TTF-1/NKX2-1* gene. The transcript variant 1 (encoding the longer protein isoform) was amplified using 6 overlapping amplicons that encompass the 3 exons of its coding-region. Similarly, 6 overlapping amplicons (5 of which were common to both variants) were generated to span the 2 exons that comprise the coding region of variant 2 (which encodes a shorter protein isoform with a N-terminus distinct from isoform 1). The entire coding-region (a single exon) of *TTF-2/FOXE1* was also amplified by producing 6 overlapping amplicons. When appropriate, germline *TTF-2/FOXE1* alterations were screened in peripheral blood DNA using analogous PCR conditions. Primers were designed according to Gen-Bank sequence (GenBank accession numbers: NM_001079668 for *TTF-1/NKX2-1* transcript variant 1, NM_003317 for *TTF-1/NKX2-1* transcript variant 2, and NM_004473 for *TTF-2/FOXE1*). The primer pairs used to generate each amplicon, as well as the annealing temperature and the amplicon expected length, are described in Table 1. PCR purified products were directly sequenced using Big Dye Terminator v1.1 Cycle Sequencing kit (Applied Biosystems, Foster City, CA).

### 2.5. QRT-PCR

The *TPO* and *TG* expression levels were quantified by QRT-PCR, as previously described [5], on an ABI Prism 7900HT Sequence Detection System using specific primers and TaqMan probes from the Assay_on_Demand products (Hs00174927_ml (*TPO*), Hs00794359_ml (*TG*); Applied Biosystems). Amplification reactions were performed in triplicate for each sample, and the results were normalized to endogenous *GAPDH* gene expression level (Pre-Developed TaqMan Assay Reagents; P/N 4326317E; Applied Biosystems).

### 2.6. Statistical Analysis

Statistical analysis was carried out using GraphPad Prism statistical software (San Diego, CA). When appropriate, values are expressed as mean ± SD. Statistical comparisons were made using the unpaired Student's *t*-test (two-tailed). Statistical significance was accepted at *P* < 0.05.

## 3. Results

### 3.1. Screening for RET/PTC Rearrangements, *BRAF* ^*V*600*E*^ Mutation, and TTF-1/NKX2-1 and TTF-2/FOXE1 Variants

*RET/PTC* rearrangements and the *BRAF* ^*V*600*E*^ mutation were identified in 5/18 and 2/18 PTCs, respectively (Table 2).

No *TTF-1* variants were identified in tumors or in controls. Two novel *TTF-2* variants, c.200C>G (p.A67G) and c.510C>A (p.A170A), were identified in heterozygosity (Table 2). In both cases, the variants were also present in constitutional DNA. In order to ascertain whether these variants were polymorphisms, we analyzed samples from 32 controls. The p.A170A variant was detected, in heterozygosity, in 1 control; none presented the p.A67G variant. In summary, the p.A170A variant was detected in 2/100 alleles and the p.A67G variant was detected in 1/100 alleles (18 patients + 32 control samples). The patient harboring the p.A67G variant inherited it from the father who presented a normal thyroid gland and normal thyroid function.

In addition, the allelic frequency (patients versus controls) for two *TTF-2* variants already described was as follows: c.387T>C (p.L129L)—0.83/0.74; c.825C>T (p.S275S)—0.67/0.66.

### 3.2. TPO and TG Expression Levels

**Figure 1:**
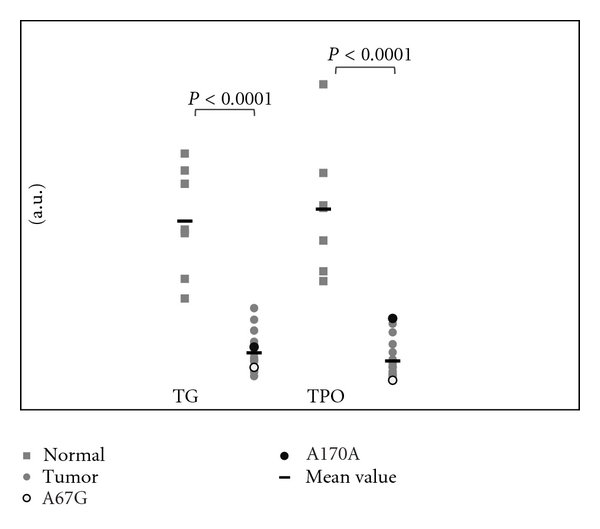
mRNA expression of *TG* and *TPO*: comparative analysis between tumors and corresponding normal thyroid tissue (possible in 7 cases, referred in Table 2 as 4, 5, 6, 8, 9, 16, and 17). Quantitative PCR results are expressed as arbitrary units (a.u.) representing *x*-fold differences relative to the reference sample.

Paired analysis of tumor and corresponding normal thyroid samples (possible in 7 cases) revealed a significant decrease (*P* < 0.0001) in *TPO* and *TG* expression among the tumor group (Figure 1), reinforcing previous results [5].

**Figure 2:**
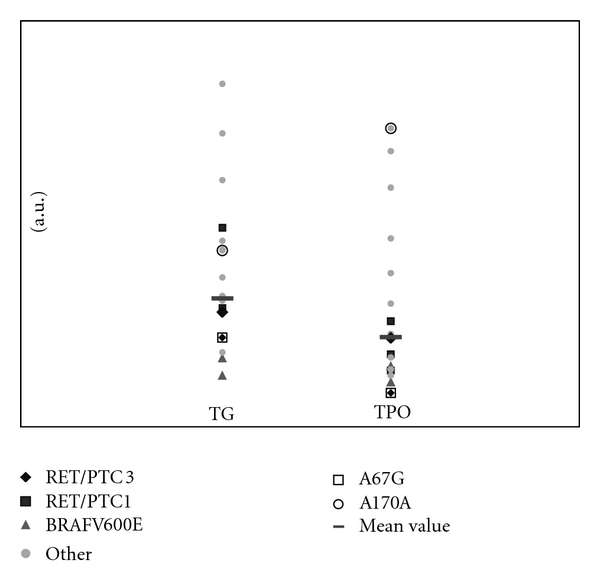
mRNA expression of *TG* and *TPO* among tumors according to the molecular alteration identified. Quantitative PCR results are expressed as arbitrary units (a.u.) representing *x*-fold differences relative to the reference sample.

Analysis of *TPO* and *TG* expression levels, among tumors, according to the subtype of molecular alteration identified, conducted to the observation that the tumor sample presenting both the *RET/PTC3* rearrangement and the p.A67G showed the lowest *TPO* expression level amongst all tumor samples (Figure 2).

## 4. Discussion

Thyroid cancer has a very low incidence in the pediatric population. The yearly incidence of DTC (PTC and FTC), in the United States, in patients from 5 to 20 years of age ranged from 0.7 to 14 cases per million, according to data in the SEER database 1975–2000 [1]. Under the age of 5, it is extremely rare [7, 8]. Exposition to ionizing radiation is a significant risk factor particularly for papillary thyroid cancer, the most common type of DTC [9, 10]. In childhood, the thyroid presents a higher susceptibility to the carcinogenetic effect of ionizing radiation than in adulthood [11]. Nonetheless, a large percentage of DTC in young patients cannot be explained by this external risk factor. 

Common variants on 9q22.33 and 14q13.3 were shown to predispose to thyroid cancer in European populations [2]. The gene nearest to the 9q22.33 locus is *FOXE1* (*TTF-2*), and *NKX2-1* (*TTF-1*) is among the genes located at the 14q13.3 locus. Almost at the same time, Landa et al. [12] showed that the variant rs1867277 within the *TTF-2* 5′UTR confers thyroid cancer susceptibility. 

We hypothesized that children and adolescents, who develop thyroid cancer, without a prior exposition to radiation and without a family history of thyroid disease, might represent a group with a higher susceptibility. Therefore, we decided to assess the potential contribution of *TTF-1* and *TTF-2* genes as sources of genetic susceptibility to thyroid cancer in this particular group. 

Eighteen patients were included in the present study. Multifocal carcinoma was found in 13/18 (72,2%), extrathyroidal extension in 10/18 (55,6%), and neck lymph node metastases in 12/18 (66,7%). Such a high frequency of multifocality, as reported in other studies [13], reinforced the hypothesis of a genetic background. 

Screening for *TTF-1* and *TTF-2* variants disclosed two novel variants in the *TTF-2* gene. One variant is a silent one, the other predicts an amino acid substitution, c.200C>G (p.A67G), located within the forkhead domain and conserved between species. The former variant was observed in 1/32 healthy individual, whereas the latter variant was not observed in the control group. However, the c.200C>G variant was also present in the patient's father who had a normal thyroid and a normal thyroid function. Moreover, the patient presenting this alteration had also a *RET/PTC3* rearrangement sufficient to justify the PTC phenotype. Thus, we considered both variants as polymorphisms. A possible interplay between the *RET/PTC3 *and the c.200C>G *TTF-2* variant cannot be excluded and awaits further clarification. The allelic frequencies of the other *TTF-2* variants were not significantly different between patients and controls. 

We assessed the functional consequences of the variants described herein by comparing the thyroglobulin (*TG*) and thyroid peroxidase (*TPO*) expression among tumors. Noteworthy, the patient with the c.200C>G variant presented the lowest *TPO* expression suggesting that this variant might alter the *TPO* transcription normally regulated by the *TTF-2* transcription factor [14]. 

Despite the small number of cases, based on present results, *TTF-1* and *TTF-2* variants do not seem to contribute greatly to an earlier age of onset of thyroid cancer. 

## 5. Conclusions

The aim of the study was to investigate whether *TTF-1* and *TTF-2* variants might contribute to thyroid cancer risk in children and adolescents. This hypothesis derived from the fact that two common variants, close from these genes, known to play an important role for thyroid organogenesis and differentiation, have been reported to be associated with a 5.7-fold increase in the risk of thyroid cancer in homozygous individuals. 

No *TTF-1* variant was identified. For *TTF-2*, 2 variants already identified had the same allelic frequency in patients and controls. Two new *TTF-2* variants (c.200C>G and c.510C>A) were identified in 2 adolescents. The variant c.510>A (p.A170A) was also identified in a control subject. The patient harboring the c.200>G variant (p.A67G) inherited it from the father who presented a normal thyroid morphology and normal thyroid function. In addition, the latter variant was associated, in the patient, with a *RET/PTC3* rearrangement sufficient to respond for the neoplastic phenotype. Altogether, results suggest that the two new *TTF-2* variants are likely to be polymorphisms. 

Noteworthy, the c.200C>G variant was associated with the lowest *TPO* expression suggesting that this variant might alter the *TPO* transcription, normally, regulated by the *TTF-2* transcription factor. Whether this occurred by chance or influenced by the *RET/PTC3* cannot be excluded. 

The frequency of *RET/PTC* rearrangements and *BRAF* mutations observed was in accordance with previous reports, thus showing that *RET/PTC* is the most common genetic alteration in papillary carcinomas from young patients. 

Although limited by the number of patients studied, our results suggest that *TTF-1* and *TTF-2* variants do not make a major contribution to an earlier onset of thyroid cancer. 

## Figures and Tables

**Table 1:** *TTF-1/NKX2-1* and *TTF-2/FOXE1*: primers and PCR conditions used.

	Amplicon	Primers (5′–3′)	Annealing temperature (°C)	Expected length (bp)
TTF-1	1sv1	agactcgctcgctcatttgt	58	231
		ctccatgcccactttcttgt		
	1sv2	gcctccactcaagccaatta	62	273
		ctccatgcccactttcttgt		
	2	tgtcgatgagtccaaagcac	62	320
		gctgttcctcatggtgtcct		
	3	ctactgcaacggcaacctg	56	404
		cctggcgcttcattttgtag		
	4	ccagcatgatccacctgac	56	222
		gtttgccgtctttcaccag		
	5	caacaggctcagcagcagt	62	317
		gaggagttcaggtgggacag		
	6	gccaggtatccagcctgtccg	60	210
		cggccaggttgttaagaaaa		

TTF-2	1	acgatcccctgagctctcc	58	301
		tgagcgcgatgtagctgtag		
	2	tggctaccgtgaaggaagag	55	300
		ggatcttgaggaagcagtcg		
	3	ggcggcatctacaagttcat	58	256
		gcatgtaagccgggtaggt		
	4	ctcggacctctccacctacc	58	300
		gaggcaaaggcgcaagag		
	5	gtcttcggcctggttcct	60	317
		gtgcgcccgtagaagtcc		
	6	gaccacggtggacttctacg	62	244
		tccattcctgttcgttctca		

**Table 2:** Demographic, histological, and molecular characteristics of study subjects. M: male; F: female; −: without alteration; +: with alteration; y: yes; n: no.

Patient	Gender	Age at diagnosis (years)	Histopathology					BRAF^V600E^ mutation	TTF1	TTF2
			Pattern	Multifocality	Extra thyroid extension	Lymph node metastases	Ret/PTC rearrangements			
1	M	5	Follicular Variant	y	y	y	PTC3	−	−	−
2	F	9	Follicular Variant	y	y	y	−	−	−	−
3	F	10	Classic	y	n	y	PTC1	−	−	−
4	F	10	Solid-Follicular Variant	n	n	n	−	−	−	−
5	F	11	Follicular Variant	y	y	y	−	−	−	−
6	F	11	Classic	y	n	y	−	+	−	−
7	M	12	Follicular Variant	y	y	y	−	−	−	−
8	M	13	Classic	y	y	y	PTC1	−	−	−
9	F	13	Follicular Variant	y	y	y	−	−	−	−
10	F	15	Multinodular Follicular Variant	y	n	n	−	−	−	A170A
11	F	15	Classic	y	y	y	−	−	−	−
12	F	16	Classic	y	y	y	PTC3	−	−	A67G
13	M	17	Follicular Variant	y	y	y	−	−	−	−
14	F	17	Encapsulated Follicular Variant	n	n	n	−	−	−	−
15	F	19	Encapsulated Follicular Variant	n	n	n	−	−	−	−
16	F	19	Encapsulated Follicular Variant	n	n	n	−	−	−	−
17	F	21	Diffuse Sclerosing	y	y	y	PTC1	−	−	−
18	F	21	Classic	n	n	n	−	+	−	−
